# Heart Rate Variability as a Prognostic Factor for Cancer Survival – A Systematic Review

**DOI:** 10.3389/fphys.2018.00623

**Published:** 2018-05-29

**Authors:** Evelyne Kloter, Katja Barrueto, Sabine D. Klein, Felix Scholkmann, Ursula Wolf

**Affiliations:** Institute of Complementary Medicine, University of Bern, Bern, Switzerland

**Keywords:** HRV, tumor, vagal nerve, malignancy, prognosis

## Abstract

An increasing cancer incidence affecting any age and social class is putting serious strain on populations and health care systems around the world. This systematic literature search aims (i) to examine the correlation of heart rate variability (HRV) and cancer patients’ prognosis, (ii) to examine the relationship of HRV and clinicopathological features, and (iii) to compare HRV between different patient groups, and between patient and control groups. We conducted a systematic literature review following the PRISMA Statement. We searched the PubMed and EMBASE databases for publications released by December 2017. The search terms were: “cancer” AND “heart rate variability” AND “human” NOT “animal” NOT “review.” A total of 19 studies were finally included in this review. Most publications were high-quality observational studies. The studies showed that higher HRV correlated positively with patients’ progression of disease and outcome. Thus, we conclude that individuals with higher HRV and advanced coping mechanisms seem to have a better prognosis in cancer progression. HRV appears to be a useful aspect to access the general health status of cancer patients.

## Introduction

With approximately 8.8 million cancer-related deaths and 14 million new cancer cases per year, at present neoplastic diseases are a significant cause of morbidity and mortality worldwide. The number of new cases of cancer is expected to rise by about 70% over the next two decades (Stewart et al., 2014) as key risk factors increase, including exposure to physical (e.g., ionizing and non-ionizing radiation) and chemical carcinogens (e.g., acrylamide, aflatoxin, endocrine disruptors), lifestyle choices (e.g., tobacco use, alcohol use, unhealthy diet, physical inactivity and circadian disruption) and infections (e.g., human papillomavirus, hepatitis B-virus, helicobacter pylori).

Clinical research spans many different fields with the aim of improving diagnosis, treatment, and prognosis through increased knowledge about cancer pathophysiology. Although guidelines support cancer care, the management of cancer patients requires predictions and decisions on an individualized rather than generalized basis, taking into account the patient’s clinicopathological and psychological situation. Hence, data on prognostic factors are integral to improve patients’ specific prognosis (Gidron et al., 2014). Prognostic factors such as tumor stage and tumor markers [e.g., prostate-specific antigen (PSA), alpha-fetoprotein (AFP), human chorionic gonadotropin (hCG)] have been shown to correlate with the course of disease and/or prognosis (Mead and Stenning, 1997; Gospodarowicz and O’Sullivan, 2003). Currently, there is much interest around such prognostic factors but their reliability remains a subject of debate. Additionally, there are many other host-related or environmental factors (e.g., pollution, nutrition) which may affect the outcome. Host-related factors include general variables such as age, sex and ethnicity, inflammatory markers (e.g., C-reactive protein) and organ functioning (forced expiratory volume in one second in lung cancer), as well as immune status and personal coping mechanisms (Moreno-Smith et al., 2010). These, together with potentially undiscovered factors, may have an impact on disease control.

Heart rate variability (HRV) is a biomarker of the autonomic nervous system (ANS) function and provides a measure of ANS through sympathetic and parasympathetic modulation of cardiac function (Karvinen et al., 2013). The vagal nerve is the main component of the parasympathetic system and the main modulator of the parasympathetic innervation of the heart. HRV analysis has already been used in recreational sports, sports medicine and other clinical fields to monitor the level of physical fitness and biofeedback procedures (Park and Thayer, 2014; Prinsloo et al., 2014; Dong, 2016; Penzel et al., 2016). HRV analysis has the potential to provide additional valuable insights into multiple physiological and pathological conditions (Malik et al., 1996). It further serves as a potential marker of stress and health in functions of an organism associated with adaptability and health (Thayer et al., 2012). In a recent review by Arab et al. (2016), chemotherapy or surgery was shown to have an important effect on HRV, which indicated an impairment of the patients’ autonomic function, i.e., an autonomic dysfunction. In another recent review that also included a meta-analysis, the overall survival of cancer patients was found to be significantly longer in a group with higher HRV compared to a group with lower HRV (Zhou et al., 2016).

The aim of this systematic literature search was to provide and appraise an overview of publications which report on the following three research questions: (i) What is the role of HRV as a biomarker and prognostic factor in cancer disease? (ii) How is HRV correlated with cancer progression? and (iii) What is the value of HRV in predicting cancer patients’ prognosis and survival outcome in comparison to different patient groups, or healthy individuals? The search focused on adult cancer patients with any kind of cancer disease whose HRV was measured during a follow-up period.

## Materials and Methods

A systematic review was conducted according to the PRISMA Statement (Moher et al., 2009). The literature search was performed on the databases PubMed and EMBASE. The search included all publications until December 2017 and was conducted independently by two of the authors (KB and EK). Only studies on humans were included. The search terms were: “cancer” AND “heart rate variability” AND “human” NOT “animal” NOT “review.”

**FIGURE 1:**
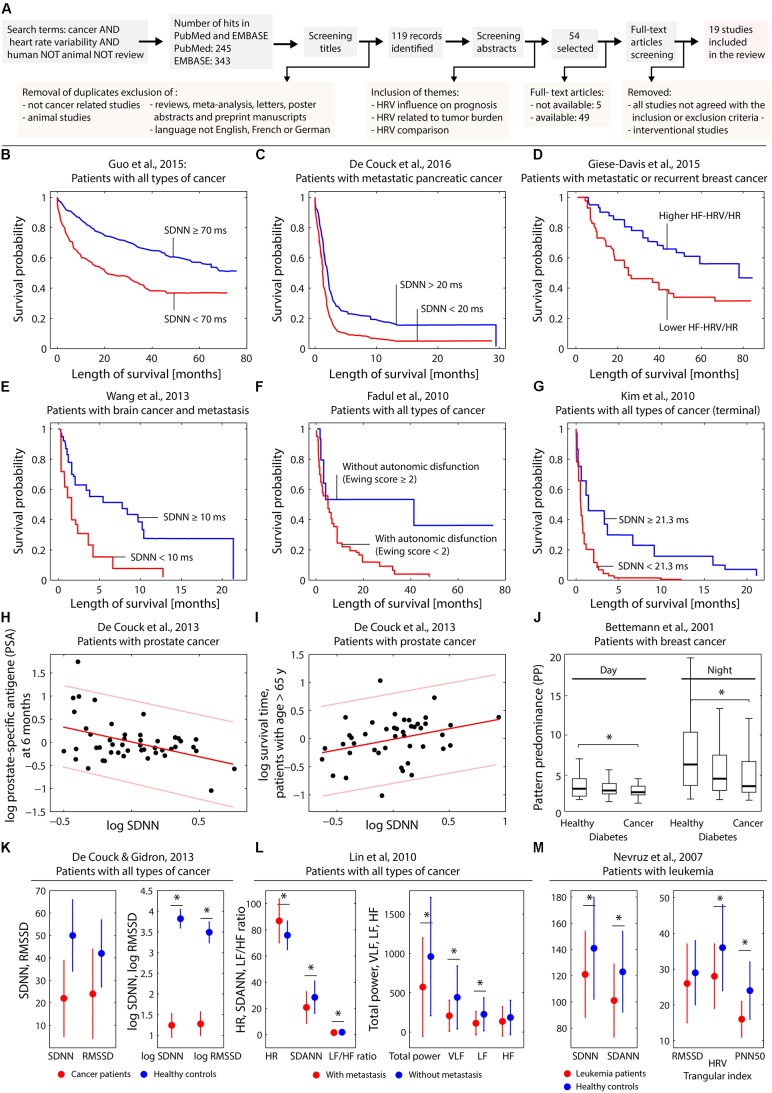
**(A)** Visualization of the search process leading to the 19 publications selected for the review. **(B–G)** Kaplan–Meier plots obtained by different studies investigating the effect of several HRV parameters on the survival probability. **(H,I)** Dependence on log SDNN on patient characteristics in case of prostate cancer. **(J)** Dependence of heart rate complexity in breast cancer patients compared to healthy controls and subjects with diabetes. **(K–M)** Differences of HRV parameters comparing healthy controls and cancer patients **(K–M)** and cancer patients with or without metastasis **(L)**. The subfigures **(B–M)** were created based on digitally extracting the numerical data from the published figures in the original published papers. For subfigure **(H)** and **(I)** the linear fit with predictions bounds was recalculated using Matlab (Natick, MA, United States). Statistically significant (*p* < 0.05) differences in the parameters comparing groups (see **J,K–M**) are indicated (^∗^).

The process of the literature search is depicted in **Figure 1A**.

### Data Collection

Publications were included when meeting the following criteria: study participants were adult cancer patients with any kind of cancer. Any number of participants and any year of publication were accepted. The prognostic factor HRV had to be assessed at least once and set in relation to other variables (e.g., progression-free survival or survival time), to prognosis or compared to healthy individuals. Meta-analysis and letters, poster abstracts or unpublished manuscripts were excluded. However, the reference lists of reviews were screened for additional publications of relevance for this systematic review.

The aim of the included studies had to be at least one of the following: (i) Investigating the correlation between HRV and patients’ prognosis, (ii) Examining the relationship of HRV with clinicopathological features, or (iii) Comparing HRV in different patient groups, or between patient and control groups.

While screening titles and abstracts, duplicates were removed. Studies were instantly excluded if they were not related to cancer patients, if the test species were not human and if the language of the publication was not English, French, or German. Publications with abstracts not related to the topic or not meeting the inclusion criteria were excluded. The full texts were read, and data extracted and appraised by two researchers (KB and EK) independently. Publications had to be available either via journals or on other scientific platforms which provided access to the Swiss University libraries. Authors were contacted if publications were not accessible through these routes. If there was no response within the period the literature search was conducted, the respective publications were excluded.

Extracted data were sorted and categorized according to the patient characteristics, HRV variables, and outcome variables. The main results are listed in **Table 1**.

**Table 1:** Summary of the 19 selected publications.

Study	Patient characteristics	Controls	HRV variables	Outcome variables	Main results
**Study topic: HRV influence on prognosis**		
Guo et al., 2015	*n* = 651 Group 1, *n* = 520, SDNN ≥ 70 ms Group 2, *n* = 131, SDNN < 70 ms	None	20- to24-h ECG monitoring (SDNN, SDANN, rMSSD, pNN50)	Survival	- Patients in group 2 had a significantly lower survival rate than patients in group 1 (25% of patients in group 2 died within 18.7 weeks vs. 78.8 weeks in group 1 patients, *p* < 0.0001)
Couck et al., 2016	*n* = 272 52.8% locally advanced pancreatic cancer; 47.2% metastatic cancer Mean age: 60.0 (±11.5) years	None	10 s ECG for HRV (time domain: SDNN, rMSSD), archival electronic records taken near diagnosis Cut-off: 20 ms (SDNN) - HighHRV > 20 ms - Low HRV < 20 ms	Survival	- SDNN significantly correlated with survival, independent of all confounders. Patients with lowSDNN survived on average only 64.05 days versus those with high SDNN who survived 133.52 days
Giese-Davis et al., 2015	*n* = 87 One group with MRBC Mean age: 54.2 ± 9.92 years, females only	None	5 min ECG for HRV (time domain: HR, frequency domain: HF)	Survival	- Higher baseline HF with significantly longer survival - Visceral metastasis status and baseline heart rate related to HF - Combined HF and HR improved survival prediction
Wang et al., 2013	*n* = 40 One group with BM from 24 NSCLC, 6 SCLC, 4 BC, 6 others: median age: 61 (39–75) years, 21 females, 19 males; patients with no previous brain OP or RT	None	5 min ECG for HRV (time domain: SDNN, rMSSD), taken before WBRT, SDNN < 10 ms or ≥ 10 ms and rMSSD < 7 ms or ≥ 7 ms usedas prognostic factors in survival analysis	Overall survival (OS)	- SDNN < 10 ms as significant independent prognosticator for OS
Chiang et al., 2013	*n* = 138 Onegroup including LC, CRC, SC, hnC, PaC, GuC, EC, others; mean age: 67.6 ± 12.2 years, 62 females, 76 males	None	5 min ECG for HRV (frequency domain: HF, TP, LF/HF), data is logarithm transformed	Survival status after 7 days	- Association between InHPF and 7-day survival in patients with non-lung cancer
Chiang et al., 2010	*n* = 33 One group with terminal HCC; mean age: 66.2 ± 13.8 years, 9 females, 24 males	None	5 min ECG for HRV (frequency domain: HF, TP)	Time to death (TTD)	- HF power significantly associated with longer TTD - Higher TP significantly associated with longer TTD - 67% accuracy of 1-week-TTD prediction for HRVand TP -73% accuracy of 2-week TTD prediction for HF, 82% for TP
Fadul et al., 2010	*n* = 47 One group with LC, GITC; median age: 59 (20–79) years, males only	None	20 min ECG for HRV (time domain: SDNN, frequency domain: VLF, LF, HF)	Survival (interval between study entry and date of death)	- Statistically significant association between survival duration in days and presence of autonomic nervous system dysfunction (i.e., ET > 2) - Trend toward significant association between survival and lower SDNN - Strong correlation between ET and SDNN, VLF - No significant association between frequency domain parameters and survival
Kim et al., 2010	*n* = 68 One group with LC, SC, EC, hbC, GC, CRC; age: 26–84 years, 34 females, 34 males	None	5 min ECG for HRV (time domain: HR, SDNN, rMSSD, frequency domain: TP, LFP, HFP)	Survival (duration, from testing until date of death)	- SDNN of 21.3 ms or less significantly associated with longer survival duration - HR greater than 100 bpm significantly associated with longer survival duration
Hoffmann et al., 2001	*n* = 35 One group with metastatic carcinoid tumor (carcinoid syndrome); mean age: 56 ± 11 years, 14 females, 21 males	None	24-h ECG for HRV (time domain: SDNN, rMSSD, pNN50)	Survival duration with follow-up of 18 ± 7 months	- Low SDNN combined with CHD with significantly higher mortality
**Study topic: HRV in relation to tumor burden**		
Gidron et al., 2014	*n* = 185 Two groups: 72 with CRC, mean age: 63.7 ± 10.8 years, 45 females, 27 males 113 with PrC, mean age 65.1 ± 8.9 years, males only	None	10 s ECG for HRV (time domain: SDNN), archival electronic records taken near diagnosis Cut-off 20 ms (SDNN) - HighHRV > 20 ms - Low HRV < 20 ms	Carcinoembryonic antigen (CEA) for CRC at 12 months Prostate specific antigen (PSA) for PrC at 6 months	Division of patients into cancer stage 1–4: - Low HRV with significantly higher CEA levels in stage 4 than in stage1 - High HRV with no significant difference between stage 4 and stage 1 CEA levels - Significant increase of CEA levels in patients with stage 4 and low HRV after 12 months (non-significant for CEA stage1–3 and low HRV) - Low HRV with significantly higher PSA levels in stage 4 than in stage2 - High HRV with insignificant difference between stage 4 and stage 2 PSA levels - Significant increase of PSA levels in patients with stage 4 andlow HRV after 6 months (insignificant for PrCstage 2,3 and low HRV)
De Couck et al., 2013	*n* = 244 Two groups: 113 with PrC, mean age: 65.06 ± 8.87 years, males only 133 with NSCLC, mean age: 62.2 ± 10.2 years (no gender specification provided)	None	10 s ECG for HRV (time domain: SDNN, rMDSS), electronic records taken near diagnosis Cut-off 20 ms (SDNN) - HighHRV > 20 ms - Low HRV < 20 ms	PrC: PSAlevels at 6 months and 2 years NSCLC: OS in full sample and survival time in deceased patients	- HRV parameters significantly predicted PSA levels at 6 months independent of confounders - HRV significantly predicted PSA levels only in stage 4 (metastatic stage), not in stage 2/3 - HRV parameters with no significant correlation to OS or survival time - Higher HRV in younger (under 65 years) NSCLC patients significantly predicted longer survival time - NSCLC patients have significantly lower SDNN than PrC patients
Mouton et al., 2012	*n* = 38 One group with CRC; mean age: 63.7 ± 10.8 years, 24 females, 14 males	None	10 s ECG for HRV (time domain: SDNN, rMDSS), archival electronic records taken near diagnosis Cut-off 20 ms (SDNN) - HighHRV > 20 ms - Low HRV < 20 ms	CEA levels at 12 months from diagnosis Examination of evolution of CEA at diagnosis, 6 and 12 months later	- Baseline SDNN significantly, inversely predicted CEA levels at 12 months, independent of confounders - Low HRV significantly predicted higher CEA levels at 12 months - rMSSD with no predictive significance - HRV-CEA relationship significant in patients with palliative treatment, not curative treatment
**Study topic: HRV comparison**		
Bijoor et al., 2016	*n* = 184 Two groups with hnC, GITC, GyC; median age : 54 (range 40–68) Early stage Group: *n* = 59 Advanced stage Group: *n* = 128	*n* = 150 Age and gender matched healthy individuals	1 min ECG for HRV (time domain: rMSSD)	HRV (rMSSD) of patients compared with healthy subjects HRV (rMSSD) of early stage compared to advanced stage group	- rMSSD significantly lower in cancer group compared to healthy group - rMSSD significantly lower in advanced stage of cancer compared- to early stage
Palma et al., 2016	*n* = 30 Two groups with BC BCG1: <18 months since breast cancer surgery; mean age 51.1 ± 8.6 BCG2: >18 months since breast cancer surgery; mean age 56.3 ± 7.4	*n* = 15 age matched healthy women; mean age 51.2 ± 10.8	30 min HR recording with Polar S810i (time domain: mean RR, rMSSD, SDNN, frequency domain: LF, HF, LF/HF)	HRV of breast cancer survivors after surgery compared to cancer free women Comparison of HRVof patients according to elapsed time since the surgery	- rMSSD, SDNN and HF are significantly reduced in post breast cancer groups compared to cancer-free group - No difference in HRV parameters regarding to the postoperative period
Koszewicz et al., 2016	*n* = 33 One group with pBT; mean age 53.0 ± 15.2	*N* = 43 Healthy volunteers, mean age 51.7 ± 10.1	HRV in% at rest (LF, HF, LF/HF)	HRV of patients compared with healthy volunteers	- HRV% significantly lower in patients group - No significant difference in LF, HF or LF/HF
De Couck and Gidron, 2013	*n* = 657 One group with PrC, PaC, CC, OC, NSCLC; mean age: 63.09 ± 11.07 years, 309 females, 348 males	One group 21’438 healthy adults for HRV-control	10 s ECG for HRV (time domain: SDNN, rMSSD), archival electronic records taken near diagnosis Cut-off 20 ms (SDNN) - HighHRV > 20 ms - Low HRV < 20 ms	HRV (indices) of patients compared withHRV of healthy subjects	- Mean HRV of cancer patients significantly lower than HRV of healthy samples - Significantly lower HRV in advanced stages (3, 4) than early stages - Lower HRV in patients with age > 65 years - HRV with tendency to significance in group < 65 - OC and NSCLC with significantly lower HRV than other cancer types
Lin and Chen, 2010	*n* = 124 Two groups with hnC, LC, BC, GITC and others Group with metastasis: *n* = 61, mean age: 62.3± 12.7 years, 36 females, 25 males Group without metastasis: *n* = 63, mean age: 52.6± 10.7 years, 35 females, 28 males	None	5 min ECG for HRV (time domain: HR, SDANN, frequency domain: TP, LF, HF, VLF, LF/HF ratio)	Comparison of HRV of patients with and without metastasis	- HR significantly higher in patients with metastasis - SDANN, TP, LF and VLF significantly lower in patients with metastasis - No statistical significance of HF and LF/HF ratio between the two groups
Nevruz et al., 2007	*n* = 36 1 group with *n* = 14 ALL, *n* = 22 AML; mean age: 34 ± 16 years, 11 females, 25 males No previous treatment	*n* = 32 age-matched healthy subjects; 9 female, 23 males, mean age: 33 ± 10 years	24-h ECG for HRV (time domain: SDNN, SDANN, HRV-triangular index, rMSSD, PNN50, SNN50) within 2 weeks of diagnosis	Comparison of HRV parameters of leukemia to control group parameters Comparison of HRV parameters of ALLto HRV parameters of AML	- SDNN, SDANN, SNN50, PNN50, HRV-triangular index values significantly lower in leukemia patients compared to control group - HRVdecreases in acute leukemias
Bettermann et al., 2001	*n* = 37 One Group B with BC;mean age: 56 ± 12 years, females only Subgroups: B1: with metastases, B0: non-metastases	Two groups Group C: *n* = 37 healthy controls; mean age 53 ± 12 years, females only Group D: *n* = 40 diabetic controls; mean age: 55 ± 15 years, females only	24-h ECGfor HRV (frequency domain: LF,HF, LF/HF, mean RR)	Comparison of HRV of B, C, D Comparison of HRV of B0, B1	- No significant difference of HRV parameters between B and C - No significant difference of HRV parameters between B and D - Lower HRV in B1 compared to B0 (non-significant)

*HR, heart rate; RR, time interval between R-peaks in the ECG, i.e., the inter-beat interval (IBI); NN, normal-to-normal intervals, i.e., intervals between normal R-peaks; SDANN, 24-h standard deviation of average NN intervals; SDNN, standard deviation of NN intervals; rMSSD, root mean square of successive differences; pNN50, percentages of differences between adjacent normal NN intervals > 50 ms; SNN50, R–R intervals exceeding 50 ms; HF, high-frequency power (0.15–0.4 Hz); LF/HF, low- to high-frequency power ratio; TP, total power; lnHFP, logarithm transformed high-frequency power; VLF, very low frequency (0.0033–0.04 Hz); ET, Ewing Test Score; OS, overall survival; TTD, time to death; CHD, carcinoid heart disease; WBRT, whole brain radiotherapy; CEA, carcinoembryonic antigen; PSA, prostate-specific antigen; BM, brain metastasis; NSCLC, non-small cell lung cancer; SCLC, small cell lung cancer; BC, breast cancer; LC, lung cancer; CRC, colorectal cancer; hnC, head and neck cancer; PaC, pancreatic cancer; guC, genitourinary cancer; EC, esophagus cancer (also; oesophagus cancer); HCC, hepatocellular carcinoma; GITC, gastrointestinal cancer; SC, stomach cancer; hbC, hepatobiliary cancer; GC, gastric cancer; PrC, prostate cancer; OC, ovarian cancer; CC, cervical carcinoma; ALL, acute lymphatic leukemia; AML, acute myeloid leukemia; MRBC, metastatic or recurrent breast cancer; GyC, gynecological cancer; pBT, primary brain tumor.*

Classification of bias and a Grading of Recommendations, Assessment, Development and Evaluations (GRADE) scoring evaluation were performed in accordance with the Cochrane guidelines (Higgins and Altman, 2008).

## Results

A total of 588 matches were screened. Following the inclusion and exclusion criteria described above, 19 studies were finally included in this systematic review. Of these 19 studies, 6 were retrospective studies and 13 prospective studies. The number of patients per study varied between 30 and 657 (mean: 154.68, median: 68 patients). HRV in each patient was described, but different indices in time or frequency domain were determined in the different studies. **Table 1** provides a description of the publications included in this systematic review. **Figures 1B–M** visualizes the key findings of the studies reviewed.

The studies included in this systematic review were performed in patients with various types of cancer, i.e., breast cancer (BC): with a total of 196 patients; prostate cancer (PrC): 113; colorectal cancer (CRC): 208; non-small cell lung cancer (NSCLC): 133; stomach and esophagus cancer: 28; bladder cancer: 3; lung cancer: 51; pancreatic cancer: 332; hepatocellular- and hepatobiliary cancer: 43; genitourinary cancer: 22; gynecologic cancers (uterus, cervix, ovary): 125; head and neck cancer: 127; brain metastasis: 40; primary brain tumor: 33; leukemia non-acute: 14, acute: 22; metastatic carcinoid tumor: 35; liver cancer: 36; gastrointestinal: 91; and sarcoma: 4. In 704 patients, the type of cancer was not defined.

In the six retrospective studies (Mouton et al., 2012; De Couck and Gidron, 2013; De Couck et al., 2013; Couck et al., 2016; Gidron et al., 2014; Guo et al., 2015), the main outcome measure was HRV in correlation with survival or tumor marker levels. Additionally, HRV of groups with and without cancer, and HRV in patients with and without metastases were compared. Within the 13 prospective studies, the main outcome measures were HRV in correlation to overall survival, time to death, 7-day survival and comparison of HRV within different groups. All studies reported at least a tendency toward higher HRV signifying a useful indicator for longer survival. This was true irrespective of measurement methods, e.g., 24-h electrocardiogram (ECG) or 10 s ECG, and regardless of HRV indices, e.g., the standard deviation of normal-to-normal interval (SDNN), high-frequency (HF) range (0.15–0.4 Hz), heart rate, the root of the mean squared differences of successive normal-to-normal interval (rMSSD).

Our first research question (i) investigated the correlation between HRV and patients’ prognosis. Giese-Davis et al. (2015), described a model of overall survival prediction. They suggested that the correlation between high-frequency power and overall survival indicates that efferent cardiac vagal activity may represent overall afferent and efferent information transfer between the vagal and the immune system and may provide early clinical prognoses in cancer patients. Guo et al. (2015), demonstrated with 24-h ECG monitoring a significantly lower survival rate in patients with SDNN < 70 ms compared to patients with SDNN > 70 ms. The same conclusion, i.e., lower SDNN associated with lower survival, was reached in the studies of Fadul et al. (2010), Kim et al. (2010), Wang et al. (2013), and also De Couck et al. (2016). Chiang et al. (2013), investigated a group of patients in a palliative care setting in their last weeks of life, although a much shorter ECG time frame (10 s to 5 min ECGs) was applied. This study also showed that patients with high-frequency power less than two dimensions had a higher risk of survival of less than 7 days.

The second research question (ii) focused on the correlation between HRV and tumor burden. Gidron, in Mouton et al. (2012), De Couck et al. (2013), and Gidron et al. (2014), analyzed data of PrC, CRC, and NSCLC patients with different cancer stages and described significantly higher tumor marker levels in patients with lower HRV.

The third research question (iii) explored the comparison between different groups of patients and healthy individuals. Among all studies related to this topic (Bettermann et al., 2001; Nevruz et al., 2007; Lin and Chen, 2010; De Couck and Gidron, 2013; Gidron et al., 2014; Bijoor et al., 2016; Koszewicz et al., 2016; Palma et al., 2016), significant differences in the HRV values were found. HRV was shown to be significantly lower in cancer patients compared to healthy individuals and also significantly lower in patients with metastases or generally with advanced stages III or IV compared to non-metastatic patients. Irrespective of the HRV measurement duration and variables, all studies showed a reduced HRV in the groups with more severely affected disease. Wang et al. (2013) and De Couck et al. (2016) evaluated the difference of patients with advanced stages compared to patients with primary and secondary stages as well as compared to healthy individuals. They reported that disease severity affects HRV, as patients in early stages showed a significantly higher vagal nerve activity than patients in later stages.

## Discussion

In recent years HRV analysis has attracted increasing interest as a diagnostic tool in cardiology. In this field, decreased HRV is a predictor of adverse outcome in myocardial infarction, sudden cardiac death and congestive heart failure (Kleiger et al., 1987; Casolo et al., 1989; Cripps et al., 1991; Ponikowski et al., 1997; La Rovere et al., 1998, 2003; Makikallio et al., 2001). HRV may also be applied as an early indication for diabetic neuropathy (De Couck et al., 2012) and is progressively employed by athletes to assess the level of physical fitness and stress coping ability (De Meersman, 1993; Chen et al., 2011; Teisala et al., 2014).

For this systematic review, all study designs including observational studies were considered. According to the GRADE score, an observational study has an evidence level between low and moderate. Since most of the publications included here were observational studies, the overall score of the studies in this review was between low and moderate. It is important to note that no strong biases were detected in any of the prospective and retrospective observational studies included, and that the quality of all publications was good.

Based on the studies included in this review, HRV may be a useful non-invasive tool to evaluate prognosis of cancer patients. However, Fadul et al. (2010), Kim et al. (2010), and Mouton et al. (2012), still consider it a poor prognosticator, particularly in patients with advanced cancer. This prognostic function of HRV, specifically for patients with metastases, has been discussed in several studies. The main hypothesis is that a lower HRV is associated with tumor growth through three pathways, i.e., inflammation, oxidative stress, and sympathetic nerve activation. De Couck et al. (2013), argued that in earlier tumor stages, commonly provided treatments such as surgery and radiotherapy are successful in reducing the tumor burden, possibly leaving less of a margin for vagal nerve activity to contribute to the process. By contrast, these treatments may have less impact in later metastatic stages, where vagal nerve activity might possibly be of even more importance. HRV-lowering effects of chemotherapy and radiotherapy appeared to be reversible with treatment cessation. This effect may therefore not be relevant for a patient’s prognosis. In a recent study by Kim et al. (2015), HRV indices were compared to other clinical variables to capture overall survival in patients with advanced NSCLC. SDNN significantly correlated with poor survival, but was not an independent prognosticator for survival. This led to the conclusion that HRV, as a stand- alone method, might be a useful tool to monitor the general wellbeing of a patient, rather than to predict overall survival. Future studies are needed to clarify these findings.

This systematic review has several limitations. The design of the six retrospective studies included might be a limiting factor since HRV may change instantaneously in stressful situations, meaning that it may be affected by a patient’s state of mind at the time of measurement. This is particularly relevant considering the fact that short-term ECGs of seconds to minutes were often measured in the context of medical consultation, a potentially stressful situation for the patient depending on the topic (e.g., diagnosis, progression of disease) addressed. The HRV measurement may be influenced by stress of the situation thus not reflecting the patient’s average HRV behavior overall. At the same time, a high HRV within the context of a medical consultation may indicate good resilience or coping abilities. Given the fact that stressful situations – like medical consultations – may influence HRV measurement, we recommend that HRV measurements in cancer patients be carried out as 24-h Holter ECG.

In five of the studies, time domain measurements of HRV, such as SDNN and rMSSD, were based on 10 s archival ECG recordings. The authors mentioned the short ECG of 10 s as a possible limitation but also referred to two studies in which HRV obtained from 10 s ECGs was found to be similar to HRV obtained from 5 and 20 min ECGs (Hamilton et al., 2004; Nussinovitch et al., 2011). Although both publications suggest a good correlation of rMSSD derived from a 10 s ECG to the rMSSD of 5 min ECG, this is not the case for SDNN. In the publication by Hamilton et al. (2004), SDNN was predictive, but to a lesser extent than rMSSD. It was concluded that it was unclear whether SDNN of 10 s ECG should be applied. Nevertheless, the authors had applied the 10 s ECG variable and defined high and low HRV with an SDNN above and below 20 ms.

Further studies are needed to clarify the correlation of HRV with cancer prognosis. It might be that individuals with advanced coping abilities have better prognosis of cancer. It would be worth testing whether patients could acquire skills to diminish acute and long-term stress reactions, supporting their healing process not only on the physiological but also the psychological level (Haurand and Aatz, 2015). Options to improve resilience could include developing the personal coping capabilities of each patient through HRV biofeedback (Karavidas et al., 2007) or enhancing vagal activity through vagal stimulating drugs (Bernik et al., 2002) or vagal nerve stimulators (Murphy and Patil, 2003). Relaxation exercises have also been found to positively affect HRV (Asher et al., 2010). In addition, physical exercise and therapeutic eurhythmy (Seifert et al., 2012) have been shown to exert an enhancing effect on HRV, which was suggested to be a goal in cancer treatment due to the association of higher HRV variables with prolonged survival in cancer patients (Niederer et al., 2013, 2015). Finally, improvement of nutrition has been shown to positively affect HRV, too (Hansen et al., 2014).

## Conclusion

This manuscript is the first to systematically compile and appraise on how HRV is associated with cancer progression and the value of HRV in predicting cancer patients’ prognosis. The majority of the studies indicate that a decreased HRV is common in cancer patients, likely reflecting autonomic dysfunction associated with the disease. Additionally, the publications reported a correlation between HRV and the progression and overall survival of cancer patients. A higher HRV is hereby associated with a better prognosis for cancer patients. HRV might be a valuable biomarker in assessing patients’ progression and outcome and further research on this topic should be conducted.

More severely affected persons exhibit lower HRV, as demonstrated in this literature search by a comparison of different patient groups or by the comparison between patients and healthy individuals. For a healthy individual, a higher level of HRV is desirable. It indicates a state of calm, relaxation and capacities to rest and regenerate. There are physical and psychological illnesses, like heart diseases, diabetes or depression, where parasympathetic activity including HRV is clearly and severely reduced (De Couck et al., 2012). Based on the literature, cancer may be added to the list of illnesses where HRV is reduced.

## Author Contributions

UW: initiation of the project and supervision of all steps. KB and EK: literature search, data extraction, and appraisal. EK, KB, SK, FS, and UW: contributed substantially to the writing and revision of the manuscript and approved its final version.

## Conflict of Interest Statement

The authors declare that the research was conducted in the absence of any commercial or financial relationships that could be construed as a potential conflict of interest.
